# Vacuum Concentration Induces Phytochemical Remodeling and Quality Transitions in a Multi-Botanical Yam-Wolfberry Beverage

**DOI:** 10.3390/molecules31213916

**Published:** 2026-10-30

**Authors:** Shu Luo, Yi Song, Zhou Zhou, Xiaoyan Xu, Pushuang Gong, Lin Tao, Xia Luo

**Affiliations:** 1 Quality Research and Drug Innovation of Traditional Chinese Medicine Key Laboratory of Sichuan Province, Sichuan Academy of Chinese Medicine Sciences, Chengdu 610041, China; luoshu@sacms.com.cn (S.L.);; 2 School of Food and Bioengineering, Chengdu University, Chengdu 610106, China

**Keywords:** drug-food homologous herb, composite beverage, vacuum concentration, non-targeted metabolomics, differential metabolites

## Abstract

Vacuum concentration is widely used to reduce water content in plant-based beverages, yet it can simultaneously enrich soluble constituents and promote selective chemical alteration. How these competing processes reshape the molecular composition and quality of complex multi-herb beverages remains poorly understood. Here, we integrated time-resolved physicochemical, taste, antioxidant and untargeted metabolomic analyses to investigate the concentration of a yam-wolfberry beverage formulated from seven food-medicine botanicals. During vacuum concentration at 60 °C, quality attributes changed non-linearly over 0–240 min, with a marked transition occurring around 180 min. Total phenolic content and DPPH radical-scavenging activity reached their highest levels at this stage, whereas prolonged processing reduced soluble protein content and umami intensity; ABTS radical-scavenging activity increased throughout concentration. Untargeted UHPLC–MS/MS analysis identified 190 differentially accumulated metabolites between the unconcentrated and 180 min samples. Relative to the theoretical 1.84-fold concentration factor, 90 metabolites showed increases exceeding passive physical enrichment, 11 were consistent with concentration-driven enrichment, and 89 decreased despite water removal. Over-representation analysis of the increased metabolites highlighted aminoacyl-tRNA biosynthesis, cyanoamino acid metabolism, D-amino acid metabolism, and phenylalanine, tyrosine and tryptophan biosynthesis. These findings indicate that vacuum concentration induces stage-dependent quality changes through the combined effects of physical enrichment and selective metabolite remodeling, offering mechanistic insights for quality-oriented processing of complex botanical beverages.

## 1. Introduction

Food processing not only influences the concentration of food constituents but also progressively alters their chemical state through mechanisms such as water removal, thermal exposure, redistribution, degradation, and matrix interactions [1,2,3]. This phenomenon is particularly pertinent to plant-based beverages composed of multiple botanical ingredients [4,5], where components such as phenolics, amino acids, organic acids, carbohydrates, proteins, lipids, and other phytochemicals may exhibit varied responses to identical processing conditions. As a result, alterations in overall quality attributes may not adequately differentiate between simple solute enrichment and concurrent chemical transformation. Therefore, comprehending how these processes collectively modify the molecular composition of complex plant-based foods is crucial for informed process design and quality control.

Vacuum concentration serves as an effective processing model for investigating this issue, as the application of reduced pressure facilitates water removal at relatively moderate temperatures [6,7], while simultaneously extending the duration of thermal exposure. Previous research has demonstrated that vacuum-assisted or reduced-pressure concentration can influence phenolic retention, color, antioxidant properties, and other quality attributes of plant-derived products [8,9,10,11,12]. Nevertheless, these studies have largely depended on physicochemical or targeted chemical analyses, thereby offering limited insight into the broader molecular remodeling that accompanies concentration. Specifically, it remains uncertain whether the alterations observed during vacuum concentration primarily result from passive enrichment due to water removal or involve selective transformation and loss of individual phytochemicals. Clarifying this distinction is crucial, as concentration can simultaneously enhance the apparent abundance of soluble constituents while promoting the transformation of thermolabile compounds.

The issue becomes more complex in multi-herb beverages, where the final chemical response emerges from the combined matrix rather than from a single botanical ingredient. This study examines a yam–wolfberry beverage formulated with seven medicinal-food homologous botanical materials [13,14,15]: yam (*Dioscorea opposita* Thunb.), wolfberry (*Lycium barbarum* L.), ginseng (*Panax ginseng* C. A. Mey.), polygonatum (*Polygonatum sibiricum* Red.), chenpi (*Pericarpium Citri Reticulatae*), poria (*Poria cocos* (Schw.) Wolf), and coix seed (*Coix lacryma-jobi* L.). According to the traditional “Jun-Chen-Zuo-Shi” formulation principles, ginseng is the sovereign herb; yam, wolfberry and poria act as minister herbs; and chenpi, coix seed and polygonatum act as assistant and courier herbs. These ingredients collectively create a chemically diverse matrix comprising carbohydrates, proteins, phenolics, organic acids, alkaloids, lipids, and other small molecules. Unlike single-ingredient formulations, such a composite matrix may exhibit processing responses that are not predictable based on the behavior of individual ingredients. The selection of the seven ingredients aimed to provide complementary matrices rather than redundant phytochemical profiles: the starchy rhizomes and sclerotium (yam, polygonatum, poria) offer a carbohydrate-rich, water-retentive matrix; wolfberry and chenpi contribute carotenoids and phenolic acids; ginseng provides ginsenosides, amino acids, and small nitrogen-containing compounds; and coix seed contributes lipids. These classes, spanning polysaccharides, carotenoids, saponins, phenolic acids, amino acids and lipids, form a matrix in which processing responses are interdependent and cannot be extrapolated from any single ingredient. However, the molecular remodeling of complex multi-herb beverages during vacuum concentration remains insufficiently characterized.

Untargeted metabolomics offers a method to address this gap by capturing coordinated changes across chemically diverse small molecules and enabling pathway-level interpretation of processing-associated metabolic patterns [1,2,3]. Metabolomics has increasingly been applied in food-processing research to characterize molecular changes associated with processing, fermentation, storage, and quality formation [1,2,3]. In particular, untargeted mass spectrometry is capable of detecting extensive alterations in the metabolite profile that are not captured by conventional measurements of total phenolics, proteins, polysaccharides, or antioxidant activity alone [2,3]. By integrating metabolomic remodeling with time-resolved physicochemical, sensory, and antioxidant assessments, it becomes possible to differentiate between general physical enrichment and selective phytochemical modification during concentration processes. The present study therefore extends this body of work in two ways: it employs an untargeted two-stage UHPLC–MS/MS analysis on a matrix of seven botanicals and introduces a concentration-factor-aware classification system that distinctly separates passive physical enrichment from selective metabolite modification. In addition, the time course is sampled at nine intervals rather than only at the starting and end points, allowing the transition to be located rather than assumed.

**Figure 1:**
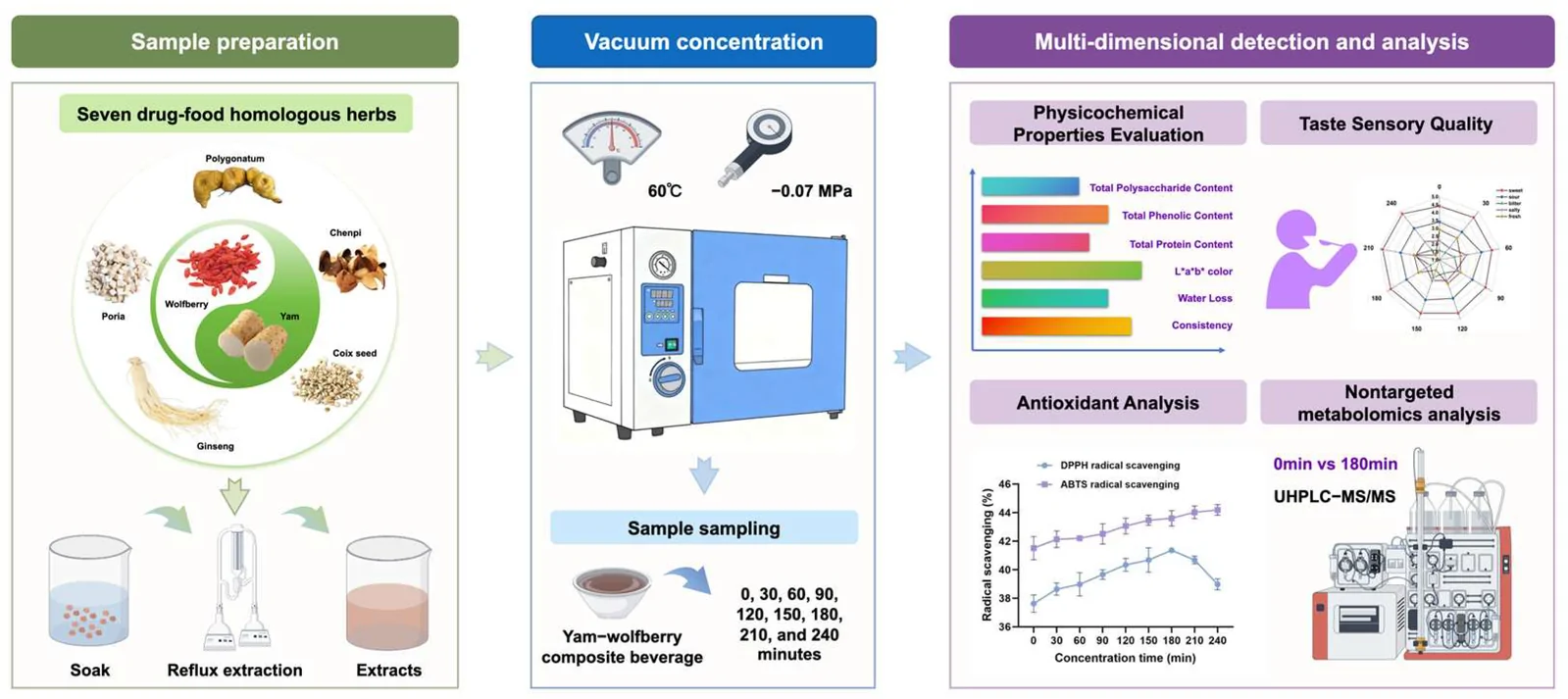
The experimental design and tests of the quality and metabolomic characteristics of yam-wolfberry composite beverage. This picture is drawn by Figdraw (www.figdraw.com).

We hypothesized that vacuum concentration would produce a non-uniform phytochemical response in the multi-herb beverage, reflecting the concurrent effects of water-driven solute enrichment and cumulative thermal exposure. We further hypothesized that this molecular remodeling would accompany a transition in sensory and antioxidant quality. To test these hypotheses, the beverage was evaluated throughout 0–240 min of vacuum concentration for physicochemical properties, basic taste attributes and DPPH and ABTS radical-scavenging activities (Figure 1). Untargeted UHPLC-MS/MS metabolomics was then used to compare the unconcentrated state (0 min) with the 180 min stage, selected because several quality attributes exhibited a marked transition around this time point. Metabolite-class distributions, concentration-factor-aware classification and KEGG pathway over-representation were integrated with the observed quality responses to determine how processing-associated molecular remodeling relates to quality formation.

## 2. Results

### 2.1. Vacuum Concentration Establishes a Progressive Quality Transition

**Figure 2:**
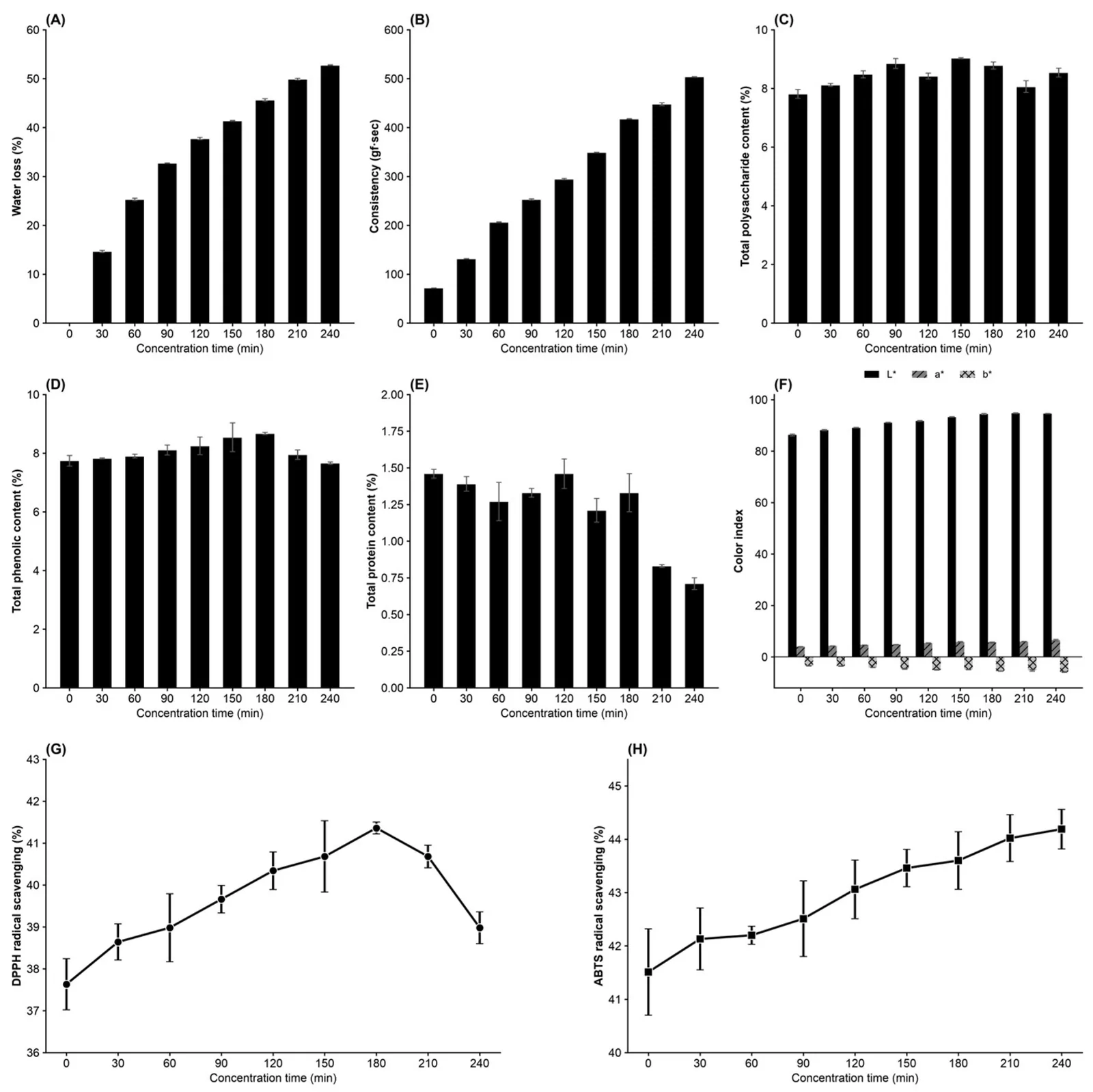
Evaluation of physicochemical and antioxidant properties of the yam–wolfberry composite beverage under different vacuum concentration durations. (**A**) Water loss rate. (**B**) Beverage consistency. (**C**) Total polysaccharide content. (**D**) Total phenolic content. (**E**) Total protein content. (**F**) Changes in color based on the L*a*b* color system. (**G**) DPPH radical-scavenging activity. (**H**) ABTS radical-scavenging activity.

The vacuum concentration process progressively modified the physical state of the beverage, as evidenced by continuous water loss and an increase in consistency over the 240 min duration (Figure 2A,B). The removal of water serves as the primary physical mechanism driving concentration by decreasing the liquid volume and augmenting the relative abundance of dissolved constituents [8,9,10,11]. Consequently, the observed increase in consistency aligns with the heightened solute concentration and diminished molecular mobility within the matrix. However, the responses of individual chemical constituents were not proportional to water loss, indicating that the process involved more than a uniform concentration of all components.

The total polysaccharide content remained relatively stable throughout the concentration process, ranging from 7.81% to 9.03% (Figure 2C). Since the assay measures the concentration of the precipitated polysaccharide fraction rather than its molecular-weight distribution or structural integrity, the data cannot ascertain whether subtle structural changes occurred. Moreover, an unchanged concentration during evaporation does not demonstrate preservation of the total recoverable amount, because the liquid mass decreases simultaneously. The apparent absence of a measurable net change should therefore be interpreted as stability of the bulk polysaccharide signal rather than as evidence of structural protection or total-mass balance; a slight rise during the intermediate stage is compatible with passive concentration, followed by a return toward the initial value.

In contrast, total phenolic content (TPC) showed a clear non-linear response (Figure 2D). TPC increased progressively from 0 to 180 min and declined at the longest concentration time. During the early and intermediate stages, water removal can increase the apparent concentration of soluble phenolics, while thermal treatment may also alter their extractability [8,9,10,11]. The subsequent decline after 180 min suggests that prolonged processing increasingly offsets the enrichment effect, potentially through degradation, oxidation, polymerization [16,17] or reduced extractability of phenolic constituents. Similar processing-dependent changes in phenolic retention have been observed in plant-derived products subjected to thermal or vacuum-assisted treatment [8,9,10,11]. The temporal coincidence between the TPC transition and subsequent changes in antioxidant and sensory responses identifies the 180 min stage as an important quality transition point.

Soluble protein exhibited a different response from TPC. Protein content remained comparatively stable through 180 min but declined markedly thereafter, reaching 0.83% at 210 min and 0.71% at 240 min (Figure 2E). This late-stage decrease indicates a reduction in the protein fraction recoverable by the Bradford assay. Thermal denaturation, aggregation, precipitation or other matrix-associated changes may contribute to this response [18,19]. The temporal coincidence between protein loss and the subsequent decline in umami intensity provides a phenotypic association between changes in the soluble nitrogen-containing fraction and sensory quality.

Color parameters also changed progressively during concentration (Figure 2F; Table S1). L* increased from 86.26 to 94.58 and a* increased modestly from 3.94 to 6.56, while b* remained slightly negative (between −3.46 and −5.88). However, the browning index remained close to zero and essentially constant (between −1.32 and −0.48) throughout, indicating no detectable browning (Figure S1). This is consistent with the metabolomic data, in which the typical browning markers 5-hydroxymethylfurfural (5-HMF) and furfural did not exceed the range expected from physical enrichment (their fold changes relative to the concentration factor were 0.64 and 0.69, respectively). The visual changes therefore primarily reflect lightening and pigment concentration rather than non-enzymatic browning; reduced pressure and the moderate temperature likely limited Maillard chemistry.

The antioxidant response also revealed a stage-dependent transition during vacuum concentration (Figure 2G,H). DPPH radical-scavenging activity increased from the initial stage to 180 min and subsequently declined, broadly paralleling the trajectory of TPC. This temporal concordance is consistent with an important contribution of phenolic constituents to radical-scavenging capacity, although the assay does not establish a direct causal relationship between TPC and DPPH activity [8,10]. In contrast, ABTS radical-scavenging activity increased continuously throughout the concentration period (Figure 2H). The divergence between DPPH and ABTS responses after 180 min indicates that the antioxidant response was not governed by a single chemical pool. Whereas the decline in DPPH activity accompanied the decrease in TPC, the continued increase in ABTS activity suggests that other redox-active constituents remained enriched or became increasingly detectable during prolonged concentration.

Taken together, the time-resolved physicochemical data reveal a non-uniform quality trajectory during vacuum concentration. Water loss and consistency increased continuously, whereas polysaccharides remained relatively stable and TPC and soluble protein exhibited stage-dependent responses. Particularly, the transition observed around 180 min, characterized by maximal TPC and subsequent changes in protein and other quality attributes, suggests that the process gradually shifted from predominantly concentration-associated enrichment toward increasing compositional alteration with prolonged thermal exposure. This transition provided the phenotypic basis for examining whether corresponding remodeling occurred at the metabolite level.

### 2.2. Sensory Responses Reveal a Quality Transition

**Figure 3:**
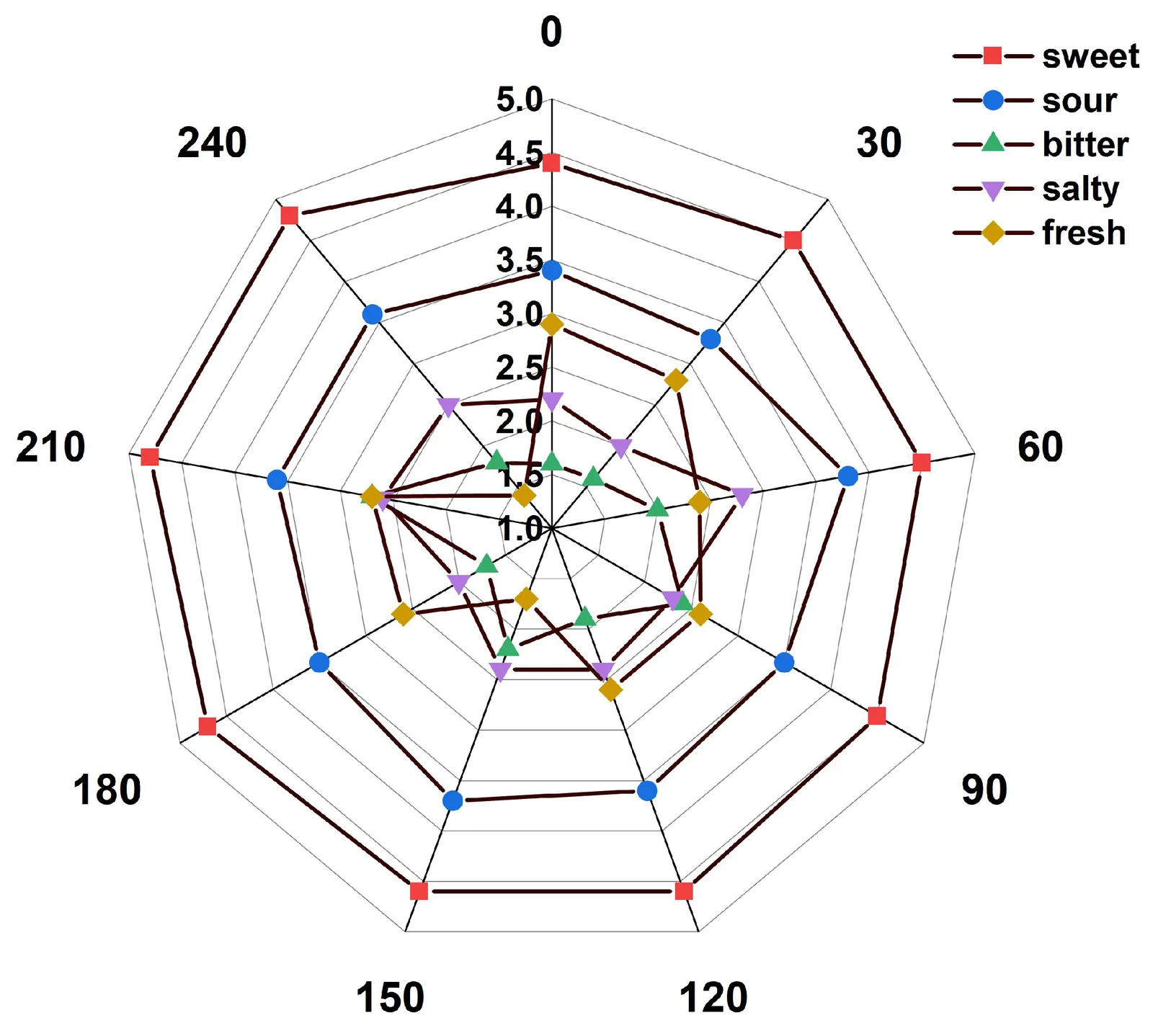
The distribution of sensory scores of the yam-wolfberry composite beverage under different vacuum concentration durations.

Vacuum concentration did not affect all taste attributes to the same extent (Figure 3). Sourness, sweetness and saltiness remained comparatively stable across the concentration period, whereas bitterness and umami exhibited clear time-dependent changes. This selective response indicates that progressive concentration altered particular taste-related chemical pools [5,20] rather than simply increasing the overall intensity of all basic tastes.

Bitterness fluctuated rather than increasing to a single maximum. It reached an early value of 2.4 at 90 min, decreased to 1.7 at 180 min, and then attained its overall maximum of 2.7 at 210 min before declining to 1.8 at 240 min. This pattern did not parallel the rise in TPC, with which bitterness was only weakly and non-significantly correlated. Phenolic compounds are recognized contributors to astringency and bitter sensory perception in plant-derived foods [21,22]. The late peak, despite continued water removal, indicates that concentration alone cannot explain the sensory trajectory, and that the availability or composition of bitter-active constituents continued to change during prolonged processing.

Umami fluctuated across the concentration period, with a transient low of 1.7 at 150 min and a recovery to 2.7 at 210 min, before falling sharply to its lowest value of 1.4 at 240 min. This late decline followed the reduction in soluble protein, suggesting an association between changes in the soluble nitrogen-containing fraction and the loss of umami perception. Glutamate and other free amino acids are established contributors to umami taste, while peptides and related nitrogen-containing compounds can also contribute to savory perception [23,24,25]. The metabolomic data further revealed changes in glutamate- and aspartate-related metabolites between 0 and 180 min, providing a molecular context for the dynamic behavior of umami.

Considered together with the physicochemical and antioxidant responses described above, the contrasting trajectories of bitterness and umami demonstrate that the quality response to vacuum concentration was multidimensional rather than a universal maximum across all quality attributes. Bitterness fluctuated and reached its highest value only late, at 210 min, whereas umami declined at the end point. Across the nine time points, TPC remained strongly correlated with DPPH activity (Spearman ρ = 0.879, *p* = 0.002), whereas TPC and bitterness (ρ = 0.360, *p* = 0.34) and ABTS activity and umami (ρ = −0.576, *p* = 0.105) were not significantly correlated. The taste attributes therefore did not vary in simple lock-step with the bulk phenolic and antioxidant measurements, indicating that selective taste-related chemical pools were remodeled independently of the bulk antioxidant response.

### 2.3. Vacuum Concentration Remodels the Phytochemical Landscape

**Figure 4:**
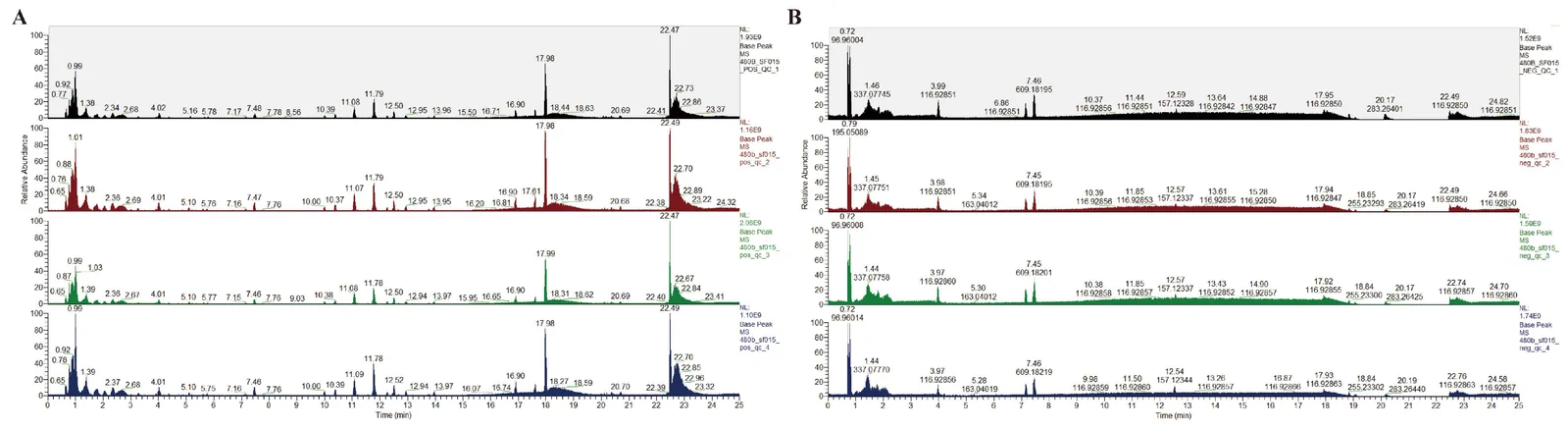
TIC diagram of the mass spectrometry detection of yam-wolfberry composite beverage samples in positive ion mode (**A**) and negative ion mode (**B**).

Untargeted UHPLC-MS/MS profiling was used to determine whether the quality transition observed at the phenotypic level was accompanied by broader remodeling of the small-molecule profile. Untargeted metabolomics is increasingly used in food-processing research because it enables simultaneous characterization of chemically diverse metabolites [26,27] and can reveal processing-associated molecular signatures beyond conventional targeted measurements [1,2,3]. Quality-control samples showed consistent total ion chromatographic profiles across injections, supporting analytical stability (Figure 4).

**Figure 5:**
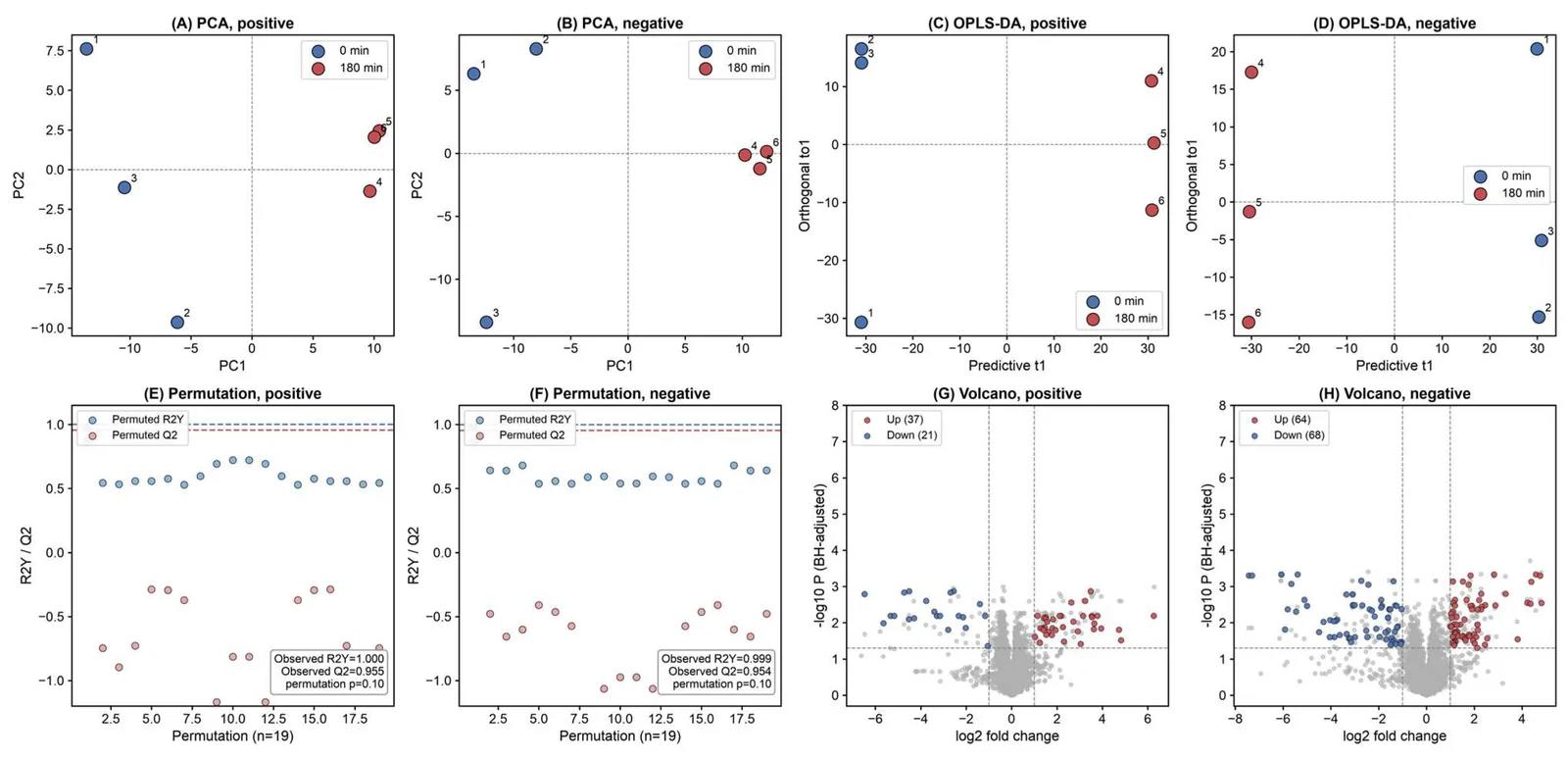
The analysis of yam-wolfberry composite beverage samples: PCA scores for metabolites in positive (**A**) and negative ion modes (**B**), OPLS-DA model scores in positive (**C**) and negative modes (**D**), OPLS-DA validation in positive (**E**) and negative modes (**F**), and volcano plots showing metabolite differences between unconcentrated and concentrated samples in positive (**G**) and negative modes (**H**). Numerals 1–6 in the score plots (**A**–**D**) label the six individual samples (three replicates per group). In the volcano plots (**G**,**H**), dashed lines mark the thresholds of |log_2_ fold change| = 1 and BH-adjusted *p* = 0.05.

After filtering, 2599 features were detected in positive-ion mode and 2649 in negative-ion mode (Table S2). PCA revealed clear separation between the 0 and 180 min groups in both ionization modes, indicating substantial differences in the detectable metabolite landscape between the two processing stages (Figure 5A,B). OPLS-DA further separated the two groups in both ionization modes (Figure 5C,D). The models showed high apparent discrimination, with R^2^Y/Q^2^ values of 1.000/0.955 in positive ion mode and 0.999/0.954 in negative ion mode (Figure 5E,F). Differential analysis identified 190 differentially accumulated metabolites (DAMs) between the 0 and 180 min stages, including 58 features in positive ion mode (37 up-regulated, 21 down-regulated) and 132 in negative ion mode (64 up-regulated, 68 down-regulated) (Figure 5G,H). The DAMs were distributed across multiple chemical classes, with organic acids and derivatives representing the largest proportion (19.13%), followed by lipids and lipid-like compounds (15.85%), alkaloids and derivatives, benzene-ring compounds, carbohydrates, and phenylpropanoids and polyketides (Table 1). The remaining seven DAMs could not be assigned to a named superclass (Table 1 note). The broad distribution of DAMs across chemical classes indicates that vacuum concentration was associated with coordinated remodeling of multiple metabolite pools rather than a response confined to a single phytochemical class.

An important consideration in interpreting these metabolomic changes is the contribution of physical concentration. Metabolomic samples were prepared from equal sample volumes rather than normalized to dry matter. At 180 min, the measured water loss was approximately 45.7%, corresponding to an estimated concentration factor of approximately 1.84-fold. To distinguish passive enrichment from selective chemical alteration, the 190 DAMs were classified according to their fold change (FC) relative to this concentration factor (Table S3). Metabolites with FC within 0.8–1.2 times the concentration factor (1.47  ≤  FC  ≤  2.21) were considered compatible with physical enrichment; those with FC  >  2.21 were classified as above-enrichment (indicating net generation, release or enhanced extractability); those with 1  ≤  FC  <  1.47 as partial degradation; and those with FC  <  1 were classified as net degradation or loss. This classification revealed that 90 DAMs (47.4%) exceeded the expected enrichment, 11 (5.8%) were compatible with passive enrichment, and 89 (46.8%) decreased despite the concentrating effect of water removal; no DAMs fell into the partial-degradation range. To test whether the absence of partial-degradation DAMs was introduced by the stringent two-fold differential threshold, the classification was applied to all 4808 QC-validated features without the threshold (Table S4). On this unfiltered basis, 177 features (3.7%) were above enrichment, 246 (5.1%) were compatible with physical enrichment, 2220 (46.2%) fell into the partial-degradation range and 2165 (45.0%) showed net degradation. Partial degradation is therefore the modal behavior of the full feature set; its apparent absence among the 190 DAMs reflects the fact that the two-fold threshold preferentially selects features with the largest fold changes. The DAM classification (90 above enrichment, 11 physical, 89 net, 0 partial) is therefore threshold-dependent and should be interpreted as a characterization of the most strongly altered features rather than of the whole metabolome.

Organic acids and derivatives constituted the largest DAM category, with 24 metabolites increased and 11 decreased after concentration. The coexistence of increased and decreased metabolites within the same chemical class further supports the view that the metabolomic response cannot be explained solely by bulk concentration. Some increases are compatible with physical enrichment, whereas decreases may reflect net loss or transformation during processing. Similar bidirectional processing-associated changes have been reported in complex plant-derived foods and beverages analyzed by untargeted metabolomics [2,3]. Comparable heterogeneity was also observed among lipid, alkaloid, carbohydrate and phenylpropanoid-related metabolites, supporting selective rather than uniform remodeling of the metabolome.

### 2.4. Amino-Acid-Centered Metabolic Remodeling Accompanies Quality Changes

**Figure 6:**
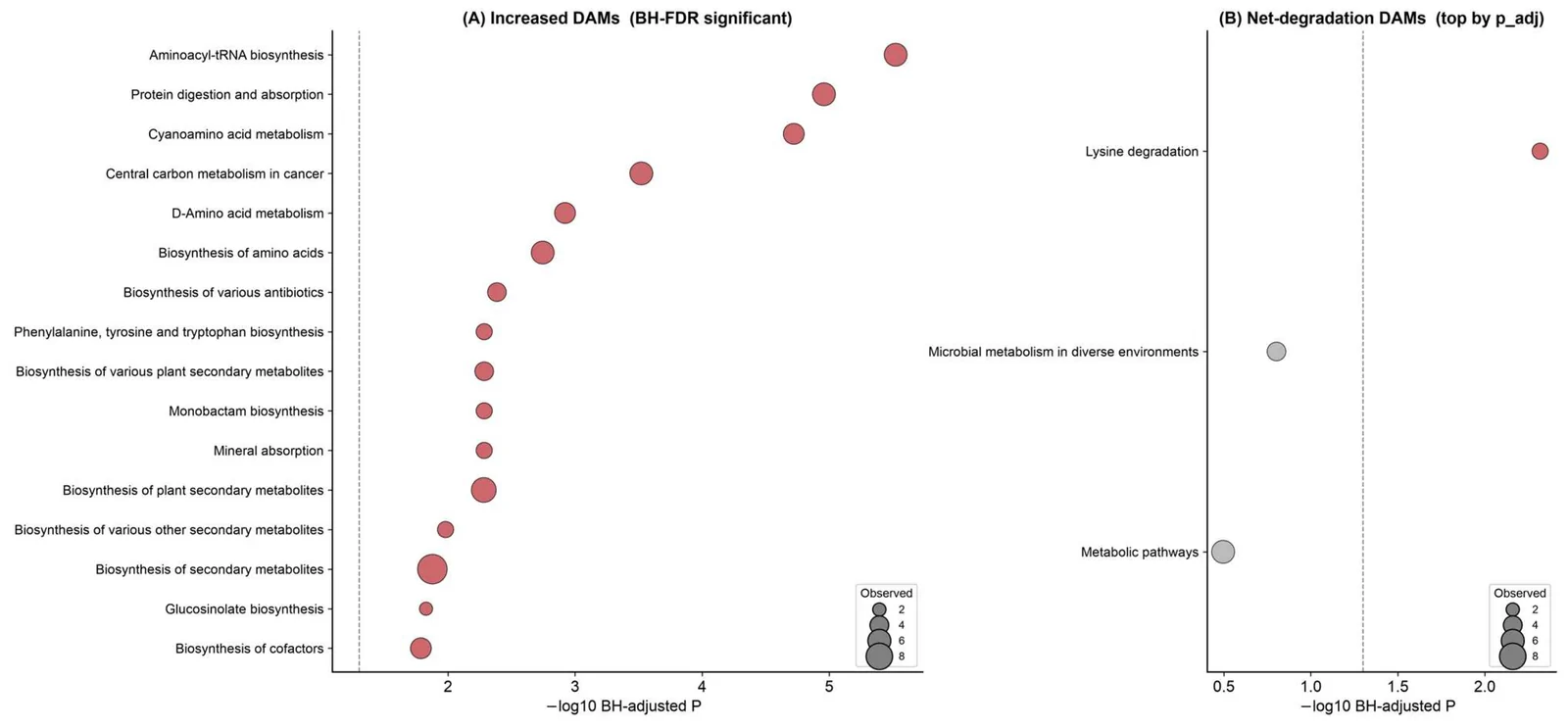
KEGG pathway over-representation of differentially accumulated metabolites. (**A**) Increased DAMs, with BH-FDR-significant pathways shown; (**B**) net-degradation DAMs, showing the three pathways with the smallest adjusted *p*-values. Dot size represents the number of observed metabolites; red dots denote BH-FDR-significant pathways and gray dots denote non-significant entries. The dashed vertical line marks the BH-adjusted *p* = 0.05 threshold.

The pathway-level analysis, conducted as an exploratory over-representation test, provided a more coherent interpretation of the broad metabolite changes. In positive ion mode, significantly enriched pathways included nicotinate and nicotinamide metabolism, alanine, aspartate and glutamate metabolism, and valine, leucine and isoleucine biosynthesis. In negative ion mode, arginine biosynthesis and alanine, aspartate and glutamate metabolism showed particularly strong enrichment, together with D-amino acid metabolism, phenylalanine, tyrosine and tryptophan biosynthesis, mannose-type O-glycan biosynthesis and phenylalanine metabolism (Figure 6). KEGG pathways were mapped for compounds with KEGG identifiers; enrichment used the observed number of mapped compounds versus the number expected from the background frequency, with Benjamini–Hochberg correction. Concentration-factor-aware KEGG enrichment of the 90 above-enrichment DAMs (21 with KEGG annotations) confirmed and extended these findings: aminoacyl-tRNA biosynthesis was the most significantly enriched pathway (6 compounds, FDR-adjusted *p* = 3.3 × 10^−6^), followed by protein digestion and absorption (6, *p* = 1.1 × 10^−5^), cyanoamino acid metabolism (5, *p* = 1.9 × 10^−5^), central carbon metabolism (6, *p* = 3.0 × 10^−4^), D-amino acid metabolism (5, *p* = 1.2 × 10^−3^) and phenylalanine, tyrosine and tryptophan biosynthesis (3, *p* = 5.2 × 10^−3^) (Table S5). In contrast, among the 89 net-degradation DAMs (10 with KEGG annotations), only lysine degradation was significantly enriched (3 compounds, *p* = 0.005). These results identify amino-acid-centered metabolism as a major pathway-level feature associated with the transition from 0 to 180 min.

To link molecular remodeling to specific quality dimensions, Spearman rank correlations were computed between each DAM and six quality attributes (TPC, soluble protein, DPPH, ABTS, bitterness and umami) across the six metabolomic samples (three per group), with Benjamini–Hochberg FDR correction (Table S6). Because metabolomic profiling was performed at only two time points, all 190 DAMs showed significant correlations (FDR < 0.05) with attributes that changed between 0 and 180 min: 101 DAMs were positively associated with TPC, DPPH and ABTS (which increased), while 89 were positively associated with protein and umami (which decreased); bitterness was essentially unchanged over the 0–180 min interval and was not used in this grouping. This analysis should therefore be interpreted as a directional association rather than an independent sample-level correlation; metabolite–quality correlation across additional processing time points is required to resolve attribute-specific associations. Nevertheless, the concordance between the direction of metabolite change and the direction of quality change provides an integrated framework for interpreting the metabolomic results.

The enrichment of amino-acid-related pathways provides a plausible molecular context for the sensory transition observed during concentration. Glutamate and other amino acids contribute directly to umami perception, while amino-acid-derived compounds and peptides participate in broader taste and flavor formation [23,24,28]. Changes in glutamate- and aspartate-related metabolites are therefore relevant to the observed umami response. Branched-chain and aromatic amino acids can also participate in thermal flavor-generation pathways, including Maillard- and Strecker-type chemistry [29,30,31]. The same metabolic remodeling may provide a molecular context for the divergence between DPPH and ABTS responses. Nicotinate/nicotinamide metabolism and arginine-related pathways were significantly enriched among the altered pathways, while ABTS scavenging continued to increase after the decline in TPC and DPPH activity. This temporal correspondence suggests that non-phenolic metabolic changes may accompany the continued ABTS response.

Taken together, the metabolomic results support a model of vacuum-concentration-induced phytochemical remodeling involving two concurrent processes: physical enrichment caused by water removal and selective alteration of individual metabolites during prolonged processing. The quality data indicate that the balance between these processes changes around the 180 min stage. At earlier stages, enrichment was associated with increasing TPC, DPPH activity and bitterness, whereas prolonged concentration was accompanied by declining TPC and soluble protein, reduced DPPH activity and later loss of umami. At the molecular level, broad metabolite remodeling and amino-acid-centered pathway changes accompanied this transition.

## 3. Discussion

Vacuum concentration is often viewed primarily as a water-removal operation, but the present results indicate that its effects on a complex plant-based beverage are more appropriately understood as a competition between physical enrichment and processing-induced chemical alteration. The continuous increase in water loss and consistency demonstrates a strong concentration effect, whereas the non-linear behavior of TPC, the late decrease in soluble protein, and the divergent DPPH and ABTS responses show that different chemical pools have distinct processing sensitivities [16,18,19]. Similar complexity has been reported for concentrated plant-derived products, in which vacuum-assisted processing can improve some quality attributes while simultaneously promoting losses or transformation of more labile constituents [8,9,10,11].

The 180 min stage is therefore better interpreted as a quality transition point than as a universal optimum. Around this stage, TPC and DPPH activity reached their highest levels, whereas bitterness peaked later, at 210 min. Subsequent processing was accompanied by declining phenolic content, soluble protein and DPPH activity, a transient late rise in bitterness, and an end-point decline in umami.

The metabolomic data further suggest that this transition involves broad redistribution of the phytochemical landscape rather than uniform enrichment. The concentration-factor-aware classification provides a particularly informative perspective: of the 190 DAMs, 90 increased beyond the 1.84-fold expected from water removal alone, 11 were compatible with passive enrichment, and 89 decreased despite the concentrating effect. The presence of both above-enrichment and net-degradation metabolites within the same chemical classes is particularly important. Because the extraction procedure used equal sample volumes without dry-matter normalization, part of the observed increase in metabolite abundance is necessarily attributable to physical concentration. A concentration-factor-aware interpretation therefore provides a more conservative framework than directly assigning every increased metabolite to biochemical formation. Conversely, decreases in relative abundance identify metabolites that were not maintained even under the concentration effect and may therefore represent processing-sensitive constituents. Future dry-weight-normalized metabolomics would allow this physical component to be quantitatively separated from chemical transformation.

Among the pathway-level findings, amino-acid-centered metabolism emerged as the most coherent molecular signature. Alanine, aspartate and glutamate metabolism were enriched in both ionization modes, while arginine biosynthesis, branched-chain amino-acid biosynthesis and aromatic amino-acid pathways were also represented. The concentration-factor-stratified KEGG enrichment further showed that these amino-acid pathways were driven primarily by above-enrichment DAMs rather than by the passive concentration effect. This pattern is biologically relevant to food quality because amino acids occupy an interface between primary metabolism, taste perception and thermal flavor chemistry [23,24,28,29]. The correspondence between amino-acid-associated remodeling and the observed sensory changes is therefore consistent with the hypothesis that changes in nitrogen-containing metabolites contribute to the quality transition. However, because free amino acids, taste-active peptides and specific reaction products were not measured across the full processing trajectory, the current evidence should be interpreted as associative rather than causal.

The antioxidant results provide a complementary perspective. The parallel temporal behavior of TPC and DPPH activity suggests that phenolic constituents make an important contribution to the early antioxidant response, whereas the continued increase in ABTS activity after the decline in TPC and DPPH indicates broader chemical contributions. This difference underscores a central advantage of metabolomics in complex food matrices: bulk antioxidant assays capture integrated chemical properties but cannot identify which metabolite pools are responsible for changes in activity [32,33]. The current data therefore support the use of metabolite–quality association analysis as a next step for identifying candidate compounds linked to specific antioxidant and sensory phenotypes.

Overall, the findings support a general model in which vacuum concentration reshapes a complex plant-derived food matrix through the concurrent effects of dehydration-driven enrichment and selective chemical alteration. The resulting metabolic remodeling is reflected in a quality transition involving phenolic-associated antioxidant activity, protein-related umami perception and broader changes in taste and antioxidant capacity. Rather than identifying a universal optimum, the study defines approximately 180 min as a transition stage under the specific conditions tested and demonstrates how integrated metabolomic and quality measurements, including concentration-factor-aware classification, can be used to interrogate processing-associated quality formation in complex multi-herb foods.

From an industrial perspective, vacuum concentration is a standard unit operation in botanical beverage and paste manufacturing, so the process is directly transferable to production and requires no additional reagents; the principal cost drivers are concentration time and energy. The defined transition stage around 180 min provides a practical stopping criterion that balances phytochemical retention against the additional energy and quality loss of prolonged processing, supporting scalability and batch consistency. At this stage, the beverage retains the highest total phenolic content and DPPH activity together with acceptable sensory scores, whereas further processing reduces soluble protein and umami. Shelf-life stability and a formal cost analysis would require dedicated studies and are not claimed here.

Several limitations should be acknowledged. First, although three independent production batches were independently extracted, concentrated and measured at each time point, the number of independent batches is limited to three, and the nine time points within each batch were obtained from a single continuous run; the repeated-measures model therefore estimates within-batch time effects with limited batch-level power, and a larger number of independent batches would be required to quantify batch-to-batch variability robustly and to support generalizable process-level inference. Second, metabolomic samples were extracted by equal volume rather than equal dry matter, so a physical enrichment component cannot be quantitatively removed; the concentration-factor classification provides a conservative framework but is not a substitute for dry-weight-normalized measurement. Third, only two time points were compared by untargeted metabolomics, and the six-sample design limits the resolution of permutation validation and the power of metabolite–quality correlation; intermediate time points would be needed to establish processing trajectories. Fourth, metabolomic fold changes were derived from per-sample median-normalized data rather than absolute concentrations established using authentic reference standards. Moreover, dilution linearity, recovery, and matrix effects were not systematically validated, and metabolite identifications ranged from confirmed to tentative. These limitations define the scope of the conclusions and identify larger-scale independent-batch replication, dry-weight-normalized, time-resolved and targeted quantification as priorities for future work.

## 4. Materials and Methods

### 4.1. Materials and Reagents

The seven drug-food homologous herbs were purchased from Sichuan Province Chinese Medicine Drinking Tablets Responsibility Co., Ltd. (Chengdu, China). All of these materials conformed to the quality requirements stated in the Chinese Pharmacopoeia 2020 Edition IV (parts of herbs). Methanol (batch No. 67-56-1), ultrapure water (batch No. 7732-18-5), and formic acid (batch no. 64-18-6) were obtained from Thermo Fisher Scientific (Waltham, MA, USA). Acetic acid was obtained from Sigma-Aldrich (St. Louis, MO, USA) (batch No. 64-19-7); 2,2-biphenyl-1-picylhydrazyl from Shanghai McLean Biochemical Technology Co., Ltd. (Shanghai, China) (batch No. C15006562); 2,2-azino-bis(3-ethyl-benzothiazole-6-sulfonic acid) diammonium salt from Fuzhou Feijing Biotechnology Co., Ltd. (Fuzhou, China) (batch No. 20231008); glucose anhydrous from Chengdu Kelong Chemical Co., Ltd. (Chengdu, China) (batch No. 2022021401); DL-malic acid from Chengdu Kelon Chemical Reagent Factory (Chengdu, China) (batch No. 20220301); quinine hydrochloride from Shanghai Haohong Biomedical Technology Co., Ltd. (Shanghai, China) (batch No. 1103568); NaCl from Chengdu Changlian Chemical Reagent Co., Ltd. (Chengdu, China) (batch No. 20200823); and sodium L-glutamate from Lotus Health Industry Group Food Co., Ltd. (Xiangcheng, China) (lot No. 20230910). All seven herbs are included in the Chinese catalog of substances traditionally regarded as both food and Chinese medicinal materials, and their use in this beverage formulation complied with relevant Chinese food safety regulations.

### 4.2. Sample Preparation and Sampling

The seven botanicals were combined as a clinical empirical formula. The specific plant parts used were the rhizome of *Dioscorea opposita*, the ripe fruit of *Lycium barbarum*, the root and rhizome of *Panax ginseng*, the rhizome of *Polygonatum sibiricum*, the dried ripe pericarp of *Citrus reticulata*, the sclerotium of *Poria cocos* and the ripe seed of *Coix lacryma-jobi*. After soaking in ultrapure water for 60 min, the materials were subjected to reflux extraction twice for 90 min each at a solid-to-liquid ratio of 1:16 (g/mL); the extraction temperature was approximately 100 °C and 2.2 L of water was used for each extraction. The two extracts were combined and centrifuged, and the combined extract was then concentrated in a vacuum concentration dryer (DZF-6050, Shanghai Jinghong Experimental Equipment Co., Ltd., Shanghai, China) at 60 °C and a relative vacuum of −0.07 MPa. The relative density of the concentrate at 180 min was measured as 1.2 g/mL. Samples were collected at 0, 30, 60, 90, 120, 150, 180, 210 and 240 min and stored at −20 °C until analysis. All physicochemical and antioxidant determinations were therefore performed in three independent batches (*n* = 3) at each time point. For nontargeted metabolomics, the 0 min and 180 min samples from each of the three independent batches were separately extracted and analyzed (*n* = 3 independent replicates per group), together with four quality control (QC) samples per ionization mode. Because the time-course observations share a batch, the time-course data are treated as repeated measures (with batch as the subject/random effect), while the 0 and 180 min metabolomic comparison is between independent groups.

### 4.3. Physicochemical Properties Evaluation

#### 4.3.1. Total Polysaccharide Content

Total polysaccharide content was determined using the anthrone–sulfuric acid colorimetric method, with the colorimetric assay adapted from a previously reported procedure [34]. Prior to the colorimetric analysis, free sugars were removed via ethanol precipitation. Specifically, a 5 mL aliquot of each sample was evaporated to dryness in a water bath, and the resulting residue was redissolved in 5 mL of hot water. Subsequently, 75 mL of 95% ethanol was gradually added with continuous stirring, and the mixture was maintained at 4 °C for 12 h. Following centrifugation, the supernatant was discarded, and the precipitated polysaccharide fraction was redissolved in hot water. This solution was then transferred to a 50 mL volumetric flask and diluted to the mark. An appropriately diluted aliquot of this solution was combined with anthrone reagent in concentrated sulfuric acid. The anthrone reagent reacts with carbohydrate-derived products to form a blue-green complex, which was measured at 625 nm after cooling. Anhydrous D-glucose served as the standard for constructing the calibration curve [34].

#### 4.3.2. Total Phenolic Content

The Folin–Ciocalteu colorimetric method was used to determine total phenolic content. The reaction consists of the Folin–Ciocalteu reagent, sodium carbonate, and the phenolic compounds in the sample. The concentrations of standard gallic acid in the range of 0.002 to 0.016 mg/mL and their absorbances were used to plot standard curves. The absorbance was measured at 765 nm [35].

#### 4.3.3. Total Protein Content

Total protein content was estimated by the Bradford assay using Coomassie brilliant blue G250. The standard protein was bovine serum albumin (BSA) with a concentration range of 0.1 to 1 mg/mL. The absorbance was measured at 595 nm [36].

#### 4.3.4. Color

The total color difference (Δ*E*, Equation (1)) was measured with an FRU color difference meter (Shenzhen Wave Optoelectronics Technology Co., Ltd., Shenzhen, China) using illuminant D65 and a 10° observer [37]. The browning index (*BI*), a measure of brown-pigment formation, was calculated from the L*a*b* coordinates as in Equations (2) and (3).

(1) $$∆E=\sqrt{{\left({∆L}^{*}\right)}^{2}+{\left({∆a}^{*}\right)}^{2}{+\left({∆b}^{*}\right)}^{2}}$$

(2) $$\mathrm{BI}=\frac{100(x-0.31)}{0.17}$$

(3) $$x=\frac{a^{*}+1.75L^{*}}{5.645L^{*}+a^{*}-3.012b^{*}}$$

#### 4.3.5. Water Loss

The samples were weighed at 0, 30, 60, 90, 120, 150, 180, 210, and 240 min after concentration. The initial weights of samples were measured and recorded as *W*_0_, and the weight of the sample after concentration was measured and recorded as *W_n_* (*n*  =  0, 30, 60, 90, 120, 150, 180, 210, 240), respectively. The water loss was calculated by the following formula:

(4) $$\mathrm{Water loss}\left(\%\right)=\frac{\left(W_{0}-W_{n}\right)}{W_{0}}\times 100.$$

#### 4.3.6. Consistency

The consistency was measured using a Universal TA texture analyzer (Shanghai Tengba Instrument Technology Co., Ltd., Shanghai, China) in Texture Profile Analysis (TPA) mode with a P/36R cylindrical probe. The specific parameters were set as follows: pre-test speed: 1 mm/s; test speed: 1 mm/s; post-test speed: 1 mm/s; and test distance: 25.0 mm.

### 4.4. Taste Sensory Quality

Standard solutions for the five basic taste attributes were prepared using appropriate concentrations of DL-malic acid, glucose, quinine, NaCl, and sodium L-glutamate to represent sourness, sweetness, bitterness, saltiness, and freshness, respectively. The concentration gradients were as follows: DL-malic acid (0.125, 0.25, 0.5, 1.0, 2.0, 4.0 mg/mL), glucose (2.5, 5, 10, 15, 20, 25 mg/mL), quinine (0.025, 0.05, 0.1, 0.2, 0.4, 0.8 mg/mL), NaCl (0.05, 0.1, 0.15, 0.2, 0.25, 0.3 mg/mL), and sodium L-glutamate (0, 0.1, 0.2, 0.3, 0.4, 0.5 mg/mL). A total of 10 trained sensory panelists (aged 22–35 years, with no taste disorders or allergies to the beverage ingredients) were recruited. The appropriate protocols for protecting the rights and privacy of all participants were utilized during the execution of the research. The panelists scored the intensities of sour, sweet, bitter, salty, and fresh tastes on a six-point scale (1–6). Prior to evaluation, panelists were instructed to refrain from consuming foods or beverages with pronounced taste attributes and to rinse their mouths with purified water before tasting each sample. Before formal evaluation, the panel completed two training sessions to recognize the five reference tastes and to calibrate the 1–6 intensity scale. During evaluation, samples were coded with three-digit numbers and presented in a randomized order in duplicate over two separate sessions; panelists rested for 2 min and rinsed with purified water between samples. The reproducibility between the two sessions was assessed from the coefficient of variation (typically <15%), and scores are reported as the mean of the ten panelists. Because the panel performed descriptive profiling rather than difference testing, no inferential statistics were applied to the sensory scores.

### 4.5. Antioxidant Analysis

#### 4.5.1. DPPH Radical-Scavenging Assay

The antioxidant capacity was determined using the 2,2-diphenyl-1-picrylhydrazyl (DPPH) radical scavenging assay as previously described [38], with minor modifications. A 2 mL aliquot of the supernatant of the yam-wolfberry composite beverage was added to 1 mL 0.2 mmol/L DPPH ethanol solution, mixed well, and incubated in the dark at 25 °C for 30 min before measuring absorbance at 517 nm. The control consisted of 1 mL of 0.2 mmol/L DPPH ethanol solution mixed with 2 mL of the ethanol/water solvent but no sample and was processed in the same way before measuring absorbance at 517 nm. A sample-color blank containing the sample and the ethanol/water solvent but no DPPH was prepared and measured in parallel, and the sample absorbance was read against this blank. The DPPH radical scavenging rate was calculated using the following formula:

(5) $$\mathrm{DPPH radical scavenging activity}\left(\%\right)=\left[1-\left(\frac{A_{s}}{A_{c}}\right)\right]\times 100$$

where *A_s_* is the absorbance of the sample at 517 nm after subtraction of the sample-color blank, meaning that the absorbance contributed by the sample and the solvent has already been removed, and *A_c_* is the absorbance of the control at 517 nm.

#### 4.5.2. ABTS Radical-Scavenging Assay

The 2,2′-azino-bis(3-ethyl-benzothiazole-6-sulfonic acid) diammonium salt (ABTS) assay was conducted as previously described [39], with slight modifications. A solution of ABTS was prepared with distilled water to a final concentration of 7.4 mmol/L. Then, 6 mL of this ABTS solution was added to 6 mL aqueous potassium persulfate (2.6 mmol/L), mixed well, and reacted for 14 h in the dark. The ABTS solution was diluted with anhydrous ethanol until the absorbance reached 0.70  at 734 nm. Then, 3.2 mL of the diluted solution was mixed with 0.8 mL of the supernatant of the yam-wolfberry composite beverage, reacted for 6 min in the dark, and then the absorbance was measured at 734 nm. A sample-color blank containing the sample and the solvent but no ABTS was prepared and measured in parallel, and the sample absorbance was read against this blank. The ABTS free radical scavenging rate was calculated as follows:

(6) $$\mathrm{ABTS radical scavenging activity}\left(\%\right)=\left[1-\left(\frac{A_{s}}{A_{c}}\right)\right]\times 100$$

where *A_s_* is the absorbance of the sample at 734 nm after subtraction of the sample-color blank, meaning that the absorbance contributed by the sample and the solvent has already been removed, and *A_c_* is the absorbance of the control at 734 nm.

### 4.6. Nontargeted Metabolomics Analysis

#### 4.6.1. Metabolite Extraction

A 200 µL aliquot of the original sample was mixed with 10 µL internal standard (10 ppm L-2-chlorophenylalanine) and 800 µL extraction solution (methanol:acetonitrile = 1:1), mixed by vortex oscillation for 1 min, ultrasonicated for 30 min, and centrifuged (10 min, 4 °C, 9000 rpm); the supernatant was then dried under vacuum. The residue was redissolved in 200 µL of a methanol-water mixture (1:1), subjected to ultrasonic filtration, and an aliquot of the filtrate was used for analysis. A fixed volume (200 µL) of each sample was extracted. The internal standard (L-2-chlorophenylalanine) was added to monitor extraction and injection variability and to assist retention-time alignment; it was not used as the basis for fold-change calculation. Because equal sample volumes rather than equal dry weights were extracted, the reported fold changes reflect metabolite concentration per unit volume and therefore include the physical enrichment effect resulting from water removal during concentration (see Section 2.3).

Equal volumes of all samples were mixed to make a quality control (QC) sample. The blank sample was a 50% methanol aqueous solution, which was pretreated in the same way as the experimental samples.

#### 4.6.2. UHPLC-MS/MS Analysis

Chromatographic separation was performed on a Thermo UltiMate 3000 UHPLC system (Thermo Fisher Scientific, San Jose, CA, USA) using a Waters Acquity UPLC HSS T3 column (2.1 mm × 100 mm, 1.8 µm; Waters, Milford, MA, USA). In positive-ion mode, mobile phase A was 0.1% formic acid in water and mobile phase B was 0.1% formic acid in acetonitrile; in negative-ion mode, water and acetonitrile without formic acid were used. The injection volume was 1 µL and the flow rate was 0.3 mL/min. The gradient program was 0–1 min, 100% A; 1–18 min, linear from 100% to 2% A; 18–21 min, 2% A; 21–21.5 min, linear from 2% to 100% A; and 21.5–25 min, 100% A for column re-equilibration. Mass spectrometry was carried out on an Orbitrap Exploris 480 mass spectrometer (Thermo Fisher Scientific, Bremen, Germany). The ESI source parameters were spray voltage 3.2 kV, sheath gas flow 40 arb, auxiliary gas flow 8 arb and capillary temperature 275 °C. Full-scan spectra were acquired over *m*/*z* 67–1000 at a resolving power of 60,000 FWHM (at *m*/*z* 200) with automatic gain control. Data-dependent MS/MS (ddMS2) was performed at a resolving power of 15,000 FWHM, with an isolation window of 1.6 *m*/*z* and a normalized collision energy of 30; data were acquired in both positive and negative polarities.

#### 4.6.3. Data Preprocessing and Annotation

The raw data were processed with Compound Discoverer 3.1 software (Thermo Fisher Scientific). After initial screening of retention time and mass-to-charge ratio, peaks were aligned across samples with a retention-time tolerance of 0.2 min and a mass tolerance of 5 ppm. Peak extraction was performed using a mass tolerance of 5 ppm, a signal-intensity tolerance of 30%, a signal-to-noise ratio of 3, a minimum signal intensity of 100,000 and specified adduct ions; peak areas were then quantified and target ions were integrated. Molecular formulas were predicted from the precursor and fragment ions and matched against the mzCloud, mzVault and ChemSpider databases. Background ions were removed by comparison with the procedural blanks, and features detected reproducibly in the QC samples were retained. Peak areas were normalized to the per-sample median area before differential analysis, and the resulting matrix was used for identification and quantification.

### 4.7. Statistical Analysis

Metabolite annotation and pathway mapping were performed in Compound Discoverer 3.1 against the KEGG database. The processed data were analyzed by PCA and OPLS-DA, with variable importance in projection (VIP) computed for the latter. DAMs were defined by a Benjamini–Hochberg (BH) FDR-adjusted *p* < 0.05 and |log2 fold change| ≥ 1. With only six samples (three per group), permutation could resolve no *p*-values below 0.05 and the observed permutation values did not meet this threshold; the supervised models were therefore treated as exploratory, and PCA separation was used as the primary group-level evidence.

The nine time points within a batch are repeated measures. A linear mixed-effects model was fitted with concentration time as the fixed factor and production batch as a random intercept, and paired comparisons between time points were BH-adjusted; the 0 and 180 min metabolomic comparison, made between independent groups, used an unpaired *t*-test with BH correction. Time trends were additionally evaluated by Spearman correlation.

To distinguish passive physical enrichment from selective alteration, each DAM was classified by its fold change relative to the concentration factor (CF = 1.84, from 45.7% water loss at 180 min): FC within 0.8–1.2 × CF was compatible with physical enrichment, higher FC indicated over-enrichment, and lower FC indicated partial (1 ≤ FC < 1.47) or net (FC < 1) degradation; the same classification was applied to all QC-validated features. KEGG pathway over-representation was tested by Fisher’s exact test with BH correction using all annotated metabolites as background, and DAM–quality Spearman correlations were BH-adjusted.

## 5. Conclusions

Vacuum concentration induced a progressive and non-uniform transition in the physicochemical, sensory, antioxidant and metabolomic characteristics of the multi-herb yam-wolfberry beverage. Water removal and consistency increased continuously, whereas polysaccharides remained comparatively stable and phenolic and protein responses became increasingly stage-dependent. TPC and DPPH radical-scavenging activity increased through approximately 180 min before declining, while bitterness fluctuated and peaked later (210 min) and umami decreased markedly by the end of concentration. The continued increase in ABTS activity despite the late decline in TPC and DPPH further indicated that different quality dimensions responded differently to processing.

Untargeted UHPLC-MS/MS metabolomics revealed broad remodeling of the phytochemical landscape between the 0 and 180 min stages, with 190 DAMs distributed across multiple chemical classes. Concentration-factor-aware classification showed that 90 DAMs exceeded the 1.84-fold physical enrichment threshold, 11 were compatible with passive enrichment and 89 underwent net degradation. Pathway analysis consistently highlighted amino-acid-centered metabolic networks, including aminoacyl-tRNA biosynthesis, alanine, aspartate and glutamate metabolism, cyanoamino acid metabolism, D-amino acid metabolism and aromatic amino-acid pathways. The concordance between these molecular changes and the observed sensory and antioxidant transitions provides a molecular framework for interpreting quality formation during vacuum concentration.

Importantly, the observed metabolomic changes should not be interpreted solely as biochemical transformation because water removal generates a substantial physical enrichment effect. The present findings therefore support a model in which vacuum concentration reshapes beverage quality through the concurrent effects of solute enrichment and selective phytochemical alteration, with approximately 180 min representing a transition stage under the tested conditions rather than a universally defined optimum. Further studies incorporating dry-matter-normalized metabolomics, targeted quantification of key metabolites and additional metabolomic time points are needed to resolve the relative contributions of physical concentration and chemical transformation and to establish causal relationships between specific metabolites and sensory or antioxidant properties.

## Figures and Tables

**Table 1:** Metabolites showing significant differences (types and proportions) between unconcentrated and concentrated wolfberry compound beverage.

Superclass Classification	Metabolite Share/%	Up-Regulated Metabolite Species	Down-Regulated Metabolite Species
Organic Acids and Derivatives	19.13	24	11
Lipids and Lipoid Compounds	15.85	16	13
Other Compounds	14.75	15	12
Alkaloids and Derivatives	11.48	9	12
Benzene Ring Compounds	9.84	7	11
Carbohydrates	8.20	5	10
Phenylpropanoids and Polyketides	6.56	8	4
Organic Heterocyclic Compounds	4.37	5	3
Organic Oxygen Compounds	3.83	2	5
Nucleosides, Nucleotides and Analogs	3.83	3	4
Peptides	2.19	3	1

Note: 183 DAMs (97 up-regulated and 86 down-regulated) were assigned to named chemical superclasses and are listed here; shares are calculated relative to these 183 classified DAMs. Seven additional DAMs (four up-regulated and three down-regulated) could not be assigned to a named superclass and are excluded from the rows, so the total number of DAMs is 190 (101 up-regulated and 89 down-regulated), as reported in the text.

## Data Availability

The raw UHPLC–MS/MS data and metadata have been deposited in Zenodo under the record https://doi.org/10.5281/zenodo.23538724. Supplementary Tables S1–S6 containing color and browning data, annotation statistics, the concentration-factor classification, all-feature distribution, KEGG enrichment, and Spearman correlations are available as Supplementary Materials. Other data supporting the findings are available from the corresponding author upon reasonable request.

## Supplementary Materials

The following supporting information can be downloaded at: https://www.mdpi.com/article/10.3390/molecules31213916/s1, Figure S1: Browning index of the beverage during vacuum concentration; Table S1: Color and browning data; Table S2: Metabolite annotation statistics; Table S3: Concentration-factor classification of differentially accumulated metabolites; Table S4: All-feature distribution; Table S5: KEGG pathway enrichment; Table S6: Spearman correlations between differentially accumulated metabolites and quality attributes.
